# Supplementary material for: Corrupted adipose tissue endogenous myelopoiesis initiates diet-induced metabolic disease
Source: eLife. 2017 Jun 28;6:e23194. doi: 10.7554/eLife.23194 (PMC5509432; doi:10.7554/eLife.23194)
Supplement: Supplementary file 1. — DOI: http://dx.doi.org/10.7554/eLife.23194.010 [file elife-23194-supp1.docx]

**Supplementary File 1**

**Supplementary File 1a : Antibody List**

| **Name** | **Clone** | **Supplier** |
| --- | --- | --- |
| CD45-1 | A20 | BD |
| CD45.2 |  | BD |
| CD117 | 2B8 | BD |
| Lin | 145-2C11 | BD |
| Ly6A/E (Sca-1) | E13-161.7 | BD |
| CD11b | M1/70 | BD |
| F4/80 | BM8 | e-bioscience |
| CD11c | N418 | e-bioscience |
| MHCII | AF6.120.1 | BD |
| CD206 | MR5D3 | AbdSerotec |
| CD34 |  |  |
| Ki67 |  |  |

**Supplementary File 1b : Primer List**

| **Primer Sequence** | **Forward** | **Reverse** |
| --- | --- | --- |
| **IL1b** | TGCCACCTTTTGACAGTGATG | TTGGAAGCAGCCCTTCATCTT |
| **PAI1** | ACAGCCTTTGTCATCTCAGCC | CCGAACCACAAAGAGAAAGGA |
| **CCL2** | GCAGTTAACGCCCCACTCA | CCCAGCCTACTCATTGGGATCA |
| **IL6** | ACACATGTTCTCTGGGAAATCGT | CAAGTGCATCATCGTTGTTCATAC |
